# Utilities Measured by Rating Scale, Time Trade-off, and Standard Gamble: Review and Reference for Health Care Professionals

**DOI:** 10.2188/jea.12.160

**Published:** 2007-11-30

**Authors:** Takeshi Morimoto, Tsuguya Fukui

**Affiliations:** Department of Clinical Epidemiology, Kyoto University Graduate School of Medicine.

**Keywords:** health-related quality of life, utility, rating scale, standard gamble, time trade-off

## Abstract

Utility is a simple expression of health-related quality of life in individuals with different states of health. A number of studies on utility measurements were conducted and published in the past. We retrieved 164 English-language articles which appeared in 1966 through 1999 for a systematic review.The number of reports has been increasing at an accelerating pace, especially during the past decade. The most widely used method of utility measurement was time trade-off, TTO (40%), followed by rating scale, RS (31%) and standard gamble, SG (29%).The utility of chronic health status was more frequently reported as compared with acute health status (907 vs 86). Accordingly, frequently explored clinical categories were cardiology, neurology, nephrology, and gastroenterology. Specifically, coronary heart disease (52 utilities), physical disability due to neurological diseases (45 utilities), chronic renal failure (74 utilities), and colorectal cancer (29 utilities) were subject to utility measurement. Mental or social dysfunctioning accounted for only a small proportion (48 utilities). There is a strong tendency for RS to yield the lowest and SG to yield the highest values.We compiled an extensive list of the results of studies on utility as a reference for health care professionals in this field.

## INTRODUCTION

Health-related quality of life (HRQOL) is now widely recognized as one of the major health outcome indices in clinical studies, health services research, and health policy formation, thus making HRQOL itself a truly important research subject. There are many ways to measure HRQOL as related to a specific disease or individual health status^1^^)^, all of which have both strength and weakness. Among these, utility is one of the simplest ways to represent HRQOL and many studies have evaluated the utilities of various states of health or disease. The concept of health state utility was developed in the early 1970s and has now a solid body of theory and a set of compelling axioms^2^^,^^3^^)^. The utility of any health state or disease is assessed as a number between zero and one. Typically, Zero means death and one perfect health. Since the utility approach provides a single cardinal measure of HRQOL and its reliability had been verified in many studies^4^^)^, it is suitable for quantitative and statistical analysis^5^^)^. Accordingly, many researchers have used utilities for decision analysis and cost-effectiveness analysis. In fact, utility is the only way to make use of HRQOL in cost-effectiveness analysis^2^^,^^3^^)^.

A number of methods have been proposed to measure utility, of which rating scale (RS), standard gamble (SG) and time trade-off (TTO) have been used most frequently. The RS consists of a line on a page with zero at one end, equivalent to death, and one at the other, equivalent to perfect health. The subjective health status, i.e., preference, is placed on the line between zero and one in such a way that the distance from zero to the placement corresponds to the degree of preference as perceived by the subject. The SG was developed by von Neumann and Morgenstern^6^^)^. It is based on a paired comparison in which the subjects choose one of the two strategies. One strategy has two possible outcomes: perfect health with probability p and death with probability 1-p. The other strategy leads to a certain health status which is intermediate in desirability between perfect health and death. Probability p is varied until the respondent registers indifference to the two strategies, at which point the utility value is p. The TTO was developed specifically for use in health care by Torrance et al. to directly assess how long a period in a state of perfect health is equivalent to a given period of ill health^7^^)^. By converting a year in a given health status to its equivalent in a state of perfect health, the value of utility can be assessed. Unlike the RS in which the subject provides explicit preference values, the SG and the TTO methods derive preference values implicitly by basing them on the subject’s responses to decision situations. Concurrently, several studies tried to convert RS to SG or TTO and showed satisfactory results^8^^,^^9^^)^.

In spite of a growing accumulation of utility studies, we still have to elicit utility data on a given health status on our own. The reasons seem to include inherent variability among individuals, a uniqueness of health status dealt with in each study, a lack of systematic review and modification for clinical use of already accumulated utility data, and a difficulty searching for relevant studies in computerized database. In fact, the word “utility” is used in many senses in the medical literature, i.e., as an efficacy of examination, a therapeutic intervention, and a representation of HRQOL. Furthermore, utility is not registered as a Medical Subject Headings (MeSH) in the MEDLINE database, which makes it difficult to search for articles on utility. Recently, Brazier et al. published an omnibus review on the use of health status measures^10^^)^. However, their report focused on technical aspects and did not deal with utility values for diseases as elicited from a variety of subject populations.

We therefore conducted a systematic review of published utility data and listed the results as a reference for researchers in this field.

## METHODS

The MEDLINE database was searched by using the following terms: rating scal*, analog scal*, linear scal*, categorical scal*, time trade-off, time trade off, time tradeoff, standard gamble, and standard reference gamble. Since “quality adjusted life years” is registered in the MeSH of MEDLINE, we also used this term. The MEDLINE database covers articles published between January 1966 and December 1999. After the first tier of screening of retrieved abstracts for the appropriate contents, full texts were collected and reviewed. Listed reference in the relevant articles and personal files were also searched.

The inclusion criteria for our review were as follows. 1) utilities on a particular health status or disease were obtained from patients with that health status or disease, or from patients with other disease, or from healthy volunteers, 2) target health status or disease was clearly stated, 3) studies were written in English. The exclusion criteria, on the other hand, were as follows. 1) utilities were not represented by definite values, e.g., only by approximations or figures, 2) target health status was complex and determined by multiple attribute, 3) the reference point was not death or perfect health, e.g., best health or worst health, 4) there was no clear definition of zero or one for the RS. In case of duplicate measurements on the same subjects in the same studies, we used the report published earlier.

## RESULTS

### Retrieval Results

From MEDLINE search, 29044 articles were retrieved. The first tier screening found 376 relevant articles for which full texts were obtained. After a critical review of these articles and relevant articles, 164 articles were judged to meet the inclusion criteria. These articles are classified in Appendix 1 according to the disease category^11^^-^^174^^)^.

### Contributed Journals

**Figure 1.**
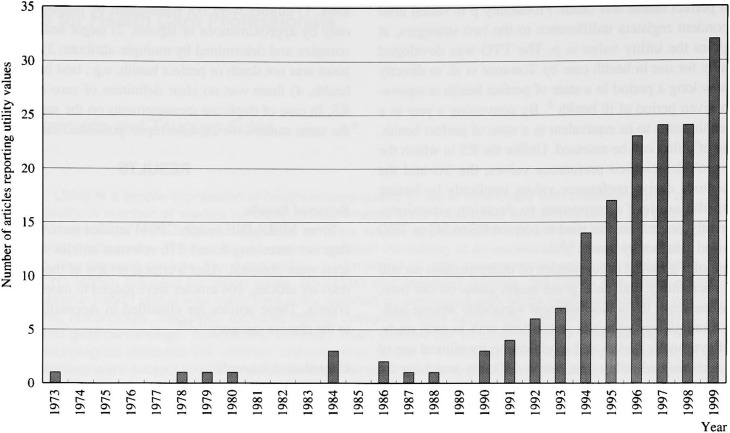
The chronological trend of studies reporting utility values in 1973-1999. The number of articles on utilities has been increasing, especially in the last decade.

**Figure 2.**
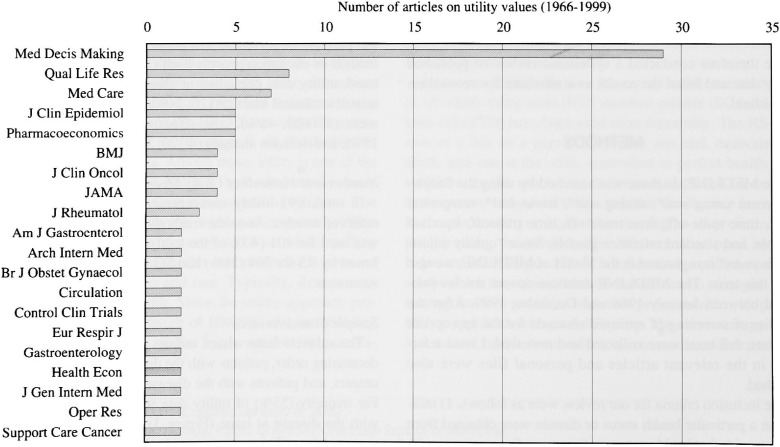
The ranking of journals in terms of the number of articles on utility values. The most frequently reporting journal was Medical Decision Making, far ahead in this field, followed by Quality of Life Research which aims to study HRQL.

The number of studies on utility has been increasing almost exponentially in the last decade (Figure 1). The most frequently reporting journal was *Medical Decision Making* (Figure 2), far ahead in this field, having published 29 utility studies so far. *Quality of Life Research*, which is designed specifically for the study of HRQOL studies, followed.

### Focus of Interest

*Medical Decision Making, Quality of Life Research* and *Medical Care* mostly published utility studies conducted for the purpose of technical assessment of utility measurement method or elicitation process itself (25/44, 57%). On the other hand, utility data published in clinical journals were mostly related to clinical trial (29/120, 24%), health outcome measurement (57/120, 48%), cost-effectiveness analysis (18/120, 15%), and decision analysis (4/120, 3%).

### Number and Methods of Utility Measurements

In total, 993 utility measurements were reported in the retrieved articles. As to the methods of eliciting utilities, TTO was used for 401 (40% of the total) utility measurements, followed by RS for 304 (31%) and SG for 288 (29%) utility measurements.

### Sample Characteristics

**Figure 3.**
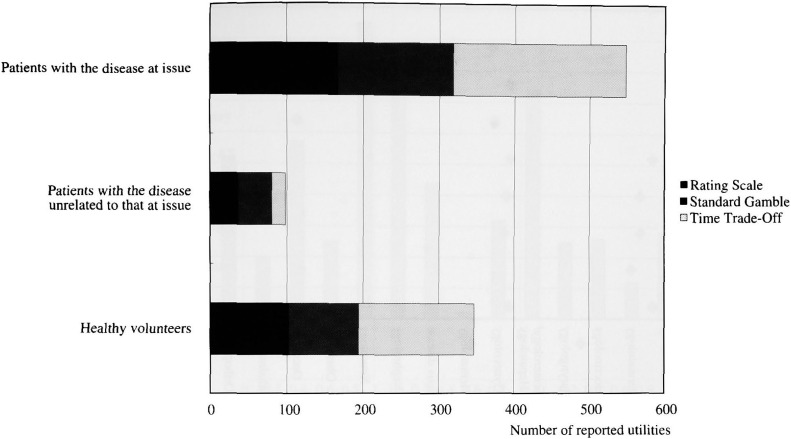
The methods of utility elicitation according to subject character. The time trade-off was most frequently used method, followed by rating scale and standard gamble. The most frequently studied subjects were patients with the disease at issue, followed by healthy volunteers and patients with the disease unrelated to that at issue.

The subjects from whom utilities were elicited included, in decreasing order, patients with the disease at issue, healthy volunteers, and patients with the disease unrelated to that at issue. Far majority (55%) of utility data were elicited from patients with the disease at issue (Figure 3). These patients were not recruited for utility measurement in the first place but rather for clinical trials. Healthy volunteers were usually medical staffs or students. Utility values obtained from patients with the disease at issue were significantly higher than those obtained from healthy volunteers or patients with an unrelated disease. For example, Ashby et al. reported that utilities of breast cancer were the highest among breast cancer patients and the lowest among healthy medical staffs and that this difference was statistically significant^151^^)^.

### Measured Health Status

**Figure 4.**
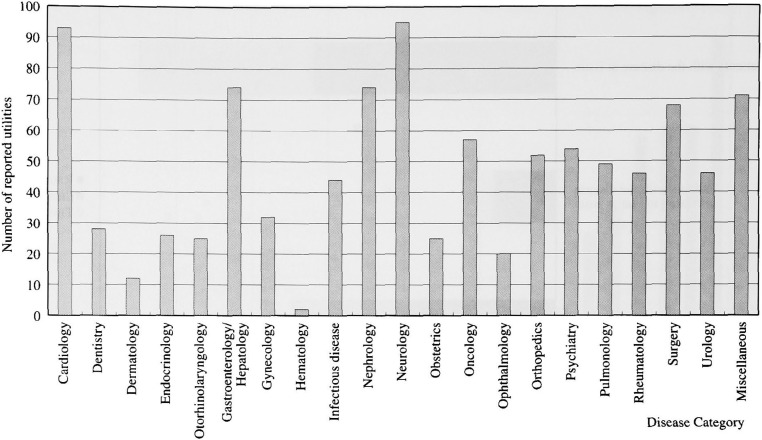
The frequency of utility elicitation according to disease category. Diseases related neurology, cardiology, nephrology and gastroenterology & hepatology were most frequently measured.

Among 993 measurements, the frequently explored clinical categories were those related to neurology, cardiology, nephrology and gastroenterology & hepatology, in decreasing order (Figure 4). Chronic health status was a dominant topic in these categories. To be concrete, they included coronary heart disease (52 utilities), physical disability due to neurological diseases (45 utilities), chronic renal failure (74 utilities), and colorectal cancer (29 utilities). In contrast, acute diseases were much less studied (86/993, 8.7%). Diseases related to mental or social dysfunctioning, such as anxiety, depression, and hospital confinement, accounted for only a minority proportion (48/993, 4.8%).

### Utility Values

**Figure 5.**
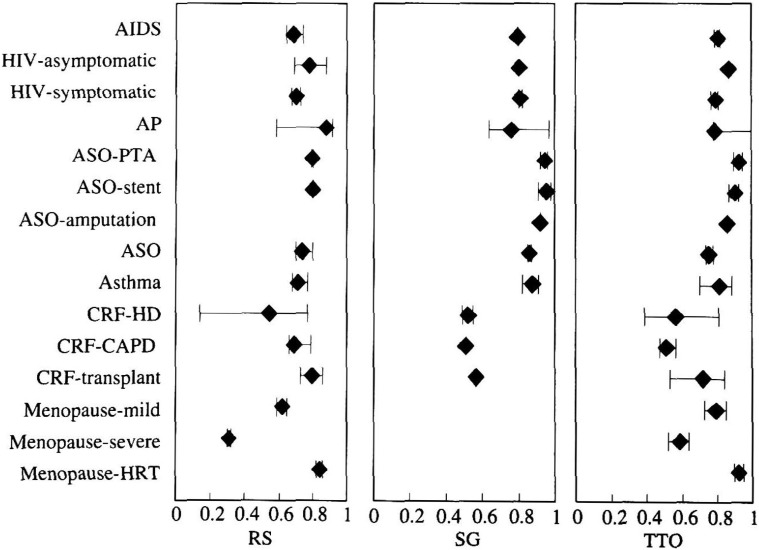
Utility values elicited from patients with the disease at issue. Rhombus shows weighted mean utilities. The horizontal bar shows the range of utility values. AIDS: acquired immunodeficiency syndrome; HIV: human immunodeficiency virus infection; AP: angina pectoris; ASO: arteriosclerosis obliterans; PTA: percutaneous transluminal angioplasty; CRF: chronic renal failure; HD: hemodialysis; CAPD: continuous ambulatory peritoneal dialysis; HRT: hormone replacement therapy; RS: rating scale; SG: standard gamble; TTO: time trade-off.

**Figure 6.**
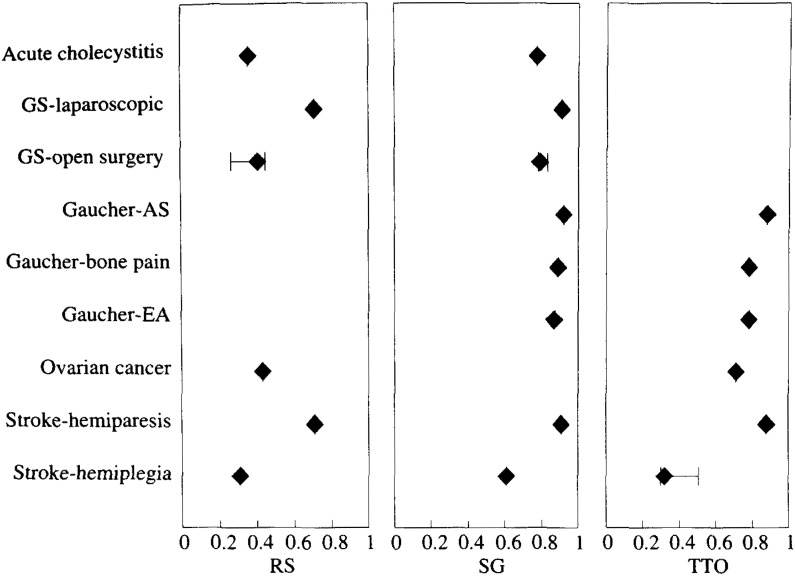
Utility values elicited from patients with the disease unrelated to that at issue. Rhombus shows weighted mean utilities. The horizontal bar shows the range of utility values. GS: gallstone; AS: asymptomatic; EA: enlarged abdomen; RS: rating scale; SG: standard gamble; TTO: time trade-off.

**Figure 7.**
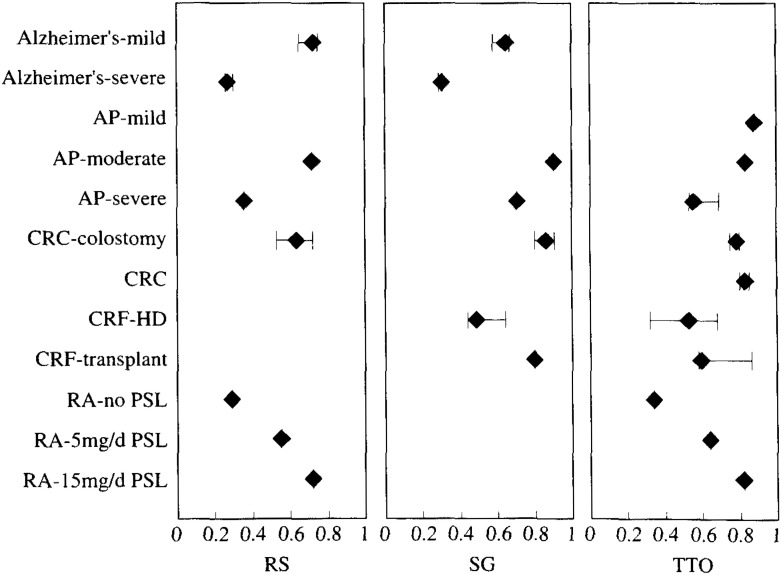
Utility values elicited from healthy volunteers. Rhombus shows weighted mean utilities. The horizontal bar shows the range of utility values. AP: angina pectoris; CRC: colorectal cancer; CRF: chronic renal failure; HD: hemodialysis; RA: rheumatoid arthritis; PSL: prednisone; RS: rating scale; SG: standard gamble; TTO: time trade-off.

Figures 5, 6, 7 and Tables 1, 2, 3 show the weighted mean, minimum, and maximum utility values for a variety of health status according to type of samples and methods of measurement. Utilities shown only as a point without a range bar were those reported in a single study. In many cases, utilities for a given health status differed if they were measured by different group of researchers. The values often depended on the subject’s characteristics, the method of measurement, and the expression of health status among others. These figures and tables show only a part of utilities listed in Appendix 1 in order to represent the range of reported utilities as many as possible (31% of the total). For example, utilities of coronary heart disease, ranging from 0.533 to 1, were frequently measured by TTO. Classified by their severity, utilities for mild disease ranged from 0.88 to 1.0, those for moderate disease 0.832 to 0.997, and those for severe disease 0.533 to 0.929. As to the relationship between measurement methods and utility values, there was a strong tendency for RS to yield the lowest and SG to yield the highest. For example. Read et al. reported that utility value corresponding to moderate angina pectoris obtained by SG was 0.93, which was significantly higher than 0.832 obtained by TTO. Furthermore, RS yielded 0.718, which was significantly lower than TTO^32^^)^.﻿

**Table 1.** Utility values elicited from patients with the disease at issue.

Diseases	Symptom	Grade	Intervention	Method	No. Subjects	Utilities		

						Max	Min	Weighted Mean
AIDS				RS	254	0.744	0.643	0.686
AIDS				SG	71	0.796	0.796	0.796
AIDS				TTO	183	0.820	0.790	0.811
HIV	Asymptomatic			RS	87	0.880	0.691	0.778
HIV	Asymptomatic			SG	40	0.804	0.804	0.804
HIV	Asymptomatic			TTO	47	0.870	0.870	0.870
HIV	Symptomatic			RS	163	0.729	0.678	0.703
HIV	Symptomatic			SG	124	0.822	0.800	0.809
HIV	Symptomatic			TTO	114	0.810	0.770	0.796
AP	Chest pain			RS	85	0.918	0.590	0.880
AP	Chest pain			SG	71	0.970	0.640	0.760
AP	Chest pain			TTO	95	1.000	0.786	0.791
ASO	Intermittent claudication		PTA	RS	212	0.800	0.790	0.796
ASO	Intermittent claudication		PTA	SG	212	0.960	0.920	0.946
ASO	Intermittent claudication		PTA	TTO	212	0.950	0.890	0.928
ASO	Intermittent claudication		Primary stent placement	RS	219	0.800	0.800	0.800
ASO	Intermittent claudication		Primary stent placement	SG	219	0.980	0.910	0.956
ASO	Intermittent claudication		Primary stent placement	TTO	219	0.930	0.870	0.909
ASO			Amputation	SG	65	0.920	0.920	0.920
ASO			Amputation	TTO	65	0.860	0.860	0.860
ASO	Intermittent claudication			RS	260	0.800	0.700	0.739
ASO	Intermittent claudication			SG	260	0.870	0.850	0.861
ASO	Intermittent claudication			TTO	260	0.780	0.740	0.758
Asthma	Dyspnea			RS	229	0.770	0.680	0.713
Asthma	Dyspnea			SG	281	0.910	0.820	0.878
Asthma	Dyspnea			TTO	115	0.890	0.706	0.816
CRF			Hemodialysis	RS	214	0.770	0.140	0.548
CRF			Hemodialysis	SG	125	0.551	0.490	0.522
CRF			Hemodialysis	TTO	997	0.810	0.390	0.566
CRF			CAPD	RS	90	0.790	0.658	0.693
CRF			CAPD	SG	66	0.510	0.510	0.510
CRF			CAPD	TTO	137	0.570	0.475	0.514
CRF			Kidney transplant	RS	139	0.860	0.730	0.798
CRF			Kidney transplant	SG	66	0.565	0.565	0.565
CRF			Kidney transplant	TTO	352	0.840	0.532	0.721
Menopause		Mild		RS	119	0.650	0.600	0.626
Menopause		Mild		TTO	119	0.850	0.730	0.794
Menopause		Severe		RS	111	0.320	0.300	0.309
Menopause		Severe		TTO	111	0.640	0.520	0.588
Menopause			HRT	RS	208	0.860	0.820	0.841
Menopause			HRT	TTO	208	0.950	0.900	0.922

AIDS: acquired immunodeficiency syndrome; HIV: human immunodeficiency virus infection; AP: angina pectoris; ASO: arteriosclerosis obliterans; PTA: percutaneous transluminal angioplasty; CRF: chronic renal failure; CAPD: continuous ambulatory peritoneal dialysis; HRT: hormone replacement therapy; RS: rating scale; SG: standard gamble; TTO: time trade-off.

**Table 2.** Utility values elicited from patients with the disease unrelated to that at issue.

Diseases	Symptom	Grade	Intervention	Method	No. Subjects	Utilities		

						Max	Min	Weighted Mean
Acute cholecystitis	RUQ pain			RS	22	0.360	0.360	0.360
Acute cholecystitis	RUQ pain			SG	16	0.770	0.770	0.770
Gallstone			Laparoscopic cholecystectomy	RS	22	0.710	0.710	0.710
Gallstone			Laparoscopic cholecystectomy	SG	16	0.910	0.910	0.910
Gallstone			Open cholecystectomy	RS	28	0.450	0.270	0.411
Gallstone			Open cholecystectomy	SG	20	0.830	0.780	0.790
Gaucher	Asymptomatic	Low blood count		SG	38	0.920	0.920	0.920
Gaucher	Asymptomatic	Low blood count		TTO	38	0.880	0.880	0.880
Gaucher	Bone pain			SG	38	0.890	0.890	0.890
Gaucher	Bone pain			TTO	38	0.780	0.780	0.780
Gaucher	Enlarged abdomen			SG	38	0.870	0.870	0.870
Gaucher	Enlarged abdomen			TTO	38	0.780	0.780	0.780
Ovarian cancer				RS	22	0.430	0.430	0.430
Ovarian cancer				TTO	22	0.710	0.710	0.710
Stroke	Hemiparesis			RS	141	0.710	0.710	0.710
Stroke	Hemiparesis			SG	141	0.910	0.910	0.910
Stroke	Hemiparesis			TTO	141	0.880	0.880	0.880
Stroke	Hemiplegia			RS	135	0.310	0.310	0.310
Stroke	Hemiplegia			SG	135	0.610	0.610	0.610
Stroke	Hemiplegia			TTO	1388	0.510	0.300	0.320

RUQ: right upper quadrant; RS: rating scale; SG: standard gamble; TTO: time trade-off.

**Table 3.** Utility values elivited from healthy volunteers.

Diseases	Symptom	Grade	Intervention	Method	No. Subjects	Utilities		

						Max	Min	Weighted Mean
Alzheimer’s disease	Dementia	Mild		RS	54	0.750	0.650	0.726
Alzheimer’s disease	Dementia	Mild		TTO	54	0.670	0.580	0.648
Alzheimer’s disease	Dementia	Severe		RS	54	0.300	0.260	0.270
Alzheimer’s disease	Dementia	Severe		TTO	54	0.310	0.290	0.305
AP	Chest pain	Mild		TTO	10	0.880	0.880	0.880
AP	Chest pain	Moderate		RS	60	0.718	0.718	0.718
AP	Chest pain	Moderate		SG	60	0.903	0.903	0.903
AP	Chest pain	Moderate		TTO	60	0.832	0.832	0.832
AP	Chest pain	Severe		RS	60	0.354	0.354	0.354
AP	Chest pain	Severe		SG	60	0.707	0.707	0.707
AP	Chest pain	Severe		TTO	70	0.690	0.533	0.555
Colorectal cancer			Colostomy	RS	99	0.719	0.527	0.636
Colorectal cancer			Colostomy	TTO	200	0.800	0.750	0.788
Colorectal cancer			Colostomy	SG	99	0.908	0.803	0.860
Colorectal cancer				TTO	200	0.850	0.800	0.829
CRF			Hemodialysis	SG	130	0.645	0.440	0.487
CRF			Hemodialysis	TTO	1121	0.678	0.320	0.526
CRF			Kidney transplant	SG	11	0.796	0.796	0.796
CRF			Kidney transplant	TTO	253	0.863	0.580	0.592
RA			No prednisone	RS	25	0.290	0.290	0.290
RA			No prednisone	TTO	25	0.340	0.340	0.340
RA			Prednisone 5mg/d	RS	25	0.550	0.550	0.550
RA			Prednisone 5mg/d	TTO	25	0.640	0.640	0.640
RA			Prednisone 15mg/d	RS	25	0.720	0.720	0.720
RA			Prednisone 15mg/d	TTO	25	0.820	0.820	0.820

AP: angina pectoris; CRF: chronic renal failure; HD: hemodialysis; RA: rheumatoid arthritis; PSL: prednisone; RS: rating scale; TTO: time trade-off; SG: standard gamble.

## DISCUSSION

The recent flourish in patient-oriented clinical research and evidence-based medicine attests to the importance of considering patients’ preferences as well as biological or life-and-death measurements. For this reason, the number of studies on utilities has been increasing almost exponentially in the last decade (Figure 1). In addition, the number of articles entitled cost-effectiveness analysis increased from seven in 1989 to 48 in 1999 in accordance with the social needs to consider cost-effectiveness in health policy as well as in clinical practice. This tendency indicates the importance for health service researchers and clinicians to be aware of HRQOL.

Our review on previously published utility studies showed that *Medical Decision Making* is the leading journal in this field (Figure 2). It reflects the leadership role of this journal in the development of methodologies related to medical decision making in the past 19 years.

Since a single cardinal measure of HRQOL, i.e., utility, is required for decision analysis and cost-effectiveness analysis, many of which were published in clinical journals^5^^)^. On the other hand, *Medical Decision Making*, *Quality of Life Research* and *Medical Care* published far less number of studies of cost-effectiveness analysis and accordingly less data on utility in spite of much data on technical assessment of utility measurement method.

We found that TTO was the most frequently used method, followed by RS and SG. This reflects the fact that RS is easy to use but subject to unreliability and that SG is difficult to use but subject to good validity^10^^)^. In this regard, TTO, going in the middle, is fairly easy to use and subject to fair validity.

Our review shows that most of the study subjects from whom utilities were elicited were patients with the disease at issue and that these patients were not recruited for utility measurement in the first place but rather for clinical trials. It reflects that patients’ preference has been widely taken into account in the assessment of outcomes of clinical trials. On the other hand, the majority of healthy volunteers were medical staffs or students. Reasons may include the convenience of access by researchers, quick apprehension of rather difficult concept of utility, and clear imagination of hypothetical clinical situations.

A chronic health status seems to be easier to imagine and assess its utility than acute health status for persons without the disease at issue. A chronic illness lasts for long time and the study subjects can exchange utility values for a given time or gamble their entire life. It is inevitable that real patients in acute distress have difficulty cooperating to answer utility inquiries.

Utility values compiled in the current study for a given health status were wide in range. This is due to the nature of the combined data for a given disease, in terms of disease stage, severity, symptoms and treatment. It is quite natural that individual patient differs from other patients with the same disease in terms of the characteristics of the disease and their own health preference. Therefore, it may be ideal to elicit utility values from particular patient when physicians conduct decision analysis and cost-effectiveness analysis in particular situation. However, comprehensively compiled data such as this could serve as a benchmark or substitute when individual utility values were not available.

We excluded multiattribute utility methods such as EuroQol, Quality of Well-Being or Health Utilities Index, from this review^2^^,^^3^^)^ because they themselves generally do not afford one-to-one corresponding utility values of a given disease and are difficult to use for a quantitative research at the present time. However, utility values of several distinct diseases were recently measured using such multiattribute utility methods and validated^175^^,^^176^^)^. Further studies are necessary in this regard.

The growing necessity of making clinical decisions based on quantitative data should further prompt utility studies in the years to come. We hope that our current review will serve as reference of utility studies ever done in the past 30 years.

## Appendix 1.

List of articles according to the disease category.

**Table:**

Author, Year	Reference	Diseases		Male(%)	Mean age (Range)	Race (Country)	
*Cardiology*							
Bosch JL, 1996	11	Arteriosclersis obliterans		70	57 (38-88)	(Netherlands)	
Bosch JL, 1998	12	Arteriosclersis obliterans		70	57 (38-88)	(Netherlands)	
Bosch JL, 1998	13	Arteriosclersis obliterans		49		(United States)	
Bosch JL, 1999	14	Arteriosclersis obliterans		72	59	(Netherlands)	
Chen AY, 1996	15	Angina		96	68	100% White	
Curry CL, 1996	16	Left ventricular hypertrophy		64	54	(International)	
de Vries SO, 1998	17	Arteriosclersis obliterans		58	61	(Netherlands)	
Fryback DG, 1993	18	Angina, Congestive heart failure, Hypertension, Myocardial infarction				100% White	
Gage BF, 1996	19	Atrial fibrillation		86	70	87% White	
Glasziou PP, 1994	20	Myocardial infarction			60 (17-87)	(Australia)	
Havranek EP, 1999	21	Congestive heart failure		59	53	58% White	
Kavanagh T, 1996	22	Congestive heart failure		81	62 (49-71)	(Canada)	
Lalonde L, 1999	23	Angina				(Canada)	
Lalonde L, 1999	24	Risk of ischemic heart disease				(Canada)	
Lawrence WF, 1996	25	Hypertension		40	67	100% White	
Molzahn AE, 1997	26	Congestive heart failure		78	48	Canada	
Moskowitz AJ, 1997	27	Congestive heart failure		84	54	84% White	
Nease RF, 1995	28	Angina				(United States)	
Nichol G, 1996	29	Angina		88	58	(Canada)	
Oldridge N, 1993	30	Myocardial infarction		88	53	(Canada)	
Pliskin JS, 1980	31	Angina			35 (27-55)	(United States)	
Read JL, 1984	32	Angina		85	39 (27-55)	(United States)	
Shephard RJ, 1998	33	Congestive heart failure		81	62		
Tsevat J, 1991	34	Post myocardial infaction				(United States)	
Tsevat J, 1993	35	Post myocardial infaction		81	60 (29-78)	(United States)	
Tsevat J, 1995	36	Post myocardial infaction		80	60	(United States)	
van Wijck EE, 1998	37	Arteriosclersis obliterans		80	65 (32-81)	(Netherlands)	

*Dentistry*							
Brickley M, 1995	38	Toothache				(United Kingdom)	
Downer, MC, 1997	39	Oral cancer		62	50	(United Kingdom)	
Fyffe HE, 1992	40	Toothache		52	(18-65)	(Scotland)	

*Dermatology*							
Bala MV, 1999	41	Herpes zoster			68	(Canada)	
Kaplan RM, 1979	42	Burn				(United States)	
Zug KA, 1995	43	Psoriasis			49 (19-78)	(United States)	

*Endocrinology*							
Douzdjian V, 1998	44	Diabetes mellitus		59		(United States)	
Fryback DG, 1993	18	Diabetes mellitus, Hyperlipidemia, Thyroid disease				100% White	
Kaplan RM, 1979	42	Obese				(United States)	
Kiberd BA, 1995	45	Diabetes mellitus				(Canada)	
Lalonde L, 1999	23	Hyperlipidemia				(Canada)	
MacKeigan LD, 1999	46	Diabetes mellitus		52	59 (27-84)	(Canada)	

*Otorhinolaryngology*							
Fryback DG, 1993	18	Sinusitis				100% White	
Llewellyn Thomas HA, 1993	47	Laryngeal cancer				(Canada)	
van der Donk J, 1995	48	Laryngeal cancer			32, 43, 62	(Netherlands)	

*Gastroenterology/Hepatology*							
Bass EB, 1994	49	Biliary disease		50	50	(United States)	
Boyd NF, 1990	50	Colorectal cancer		37	49	(Canada)	
Detsky AS, 1986	51	Total parenteral nutrition				(Canada)	
Dominitz JA, 1997	52	Colorectal cancer		99	63	(United States)	
Fryback DG, 1993	18	Colitis, Hiatal hernia. Peptic ulcer				100% White	
Irvine EJ, 1995	53	Crohn’s disease, Ulscerative colitis				(Canada)	
McLeod RS, 1991	54	Ulcerative colitis		70	34	(Canada)	
Ness RM, 1999	55	Colorectal cancer		53	54	(United States)	
Provenzale D, 1997	56	Ulcerative colitis		64	40	(United States)	
Stiggelbout AM, 1995	57	Colorectal cancer		46	62	(Netherlands)	
Tillinger W, 1999	58	Crohn’s disease		63	32	(Australia)	

*Gynecology*							
Daly E, 1993	59	Menopause		0	52.1	(United Kingdom)	
Grann VR, 1998	60	Ovarian cancer, Oophorectomy		0	38 (22-63)	(United States)	
Grann VR, 1999	61	Ovarian cancer		0	33 (20-50)	58% White	
Llewellyn Thomas HA, 1992	62	Uterus or ovarian cancer		0	58 (27-78)	(Canada)	
Sculpher M, 1998	63	Menorrhalgia		0	41	(United Kingdom)	
Sculpher MJ, 1996	64	Menorrhalgia				(United Kingdom)	
Zethraeus N, 1997	65	Menopause		0	52 (45-65)	(Sweden)	
Zethraeus N, 1999	66	Menopause		0	52 (45-65)	(Sweden)	

*Hematology*							
Lee SJ, 1997	67	Graft-versus-host disease				(United States)	
Szeinbach SL, 1999	68	Multiple myeloma		64		(United States)	

*Infectious disease*							
Bayoumi AM, 1999	69	Human immunodeficiency virus infection		92	41	(Canada)	
Johnson ES, 1996	70	Cytomegalovirus infection		99		(Australia)	
Lamping DL, 1994	71	Human immunodeficiency virus infection		93	36 (24-64)	72% White	
Law AV, 1998	72	Miscellaneous infection				(United States)	
Owens DK, 1992	73	Human immunodeficiency virus infection, Hepatitis B virus disease		64		(United States)	
Owens DK, 1997	74	Human immunodeficiency virus infection, Hepatitis B virus disease		64		(United States)	
Revicki DA, 1995	75	Human immunodeficiency virus infection		70	38	60% Black	
Sackett DL, 1978	76	Tuberculosis				(Canada)	
Torrance GW, 1973	77	Tuberculosis				(Canada)	
Tsevat J, 1992	78	Human immunodeficiency virus infection		94	35	(United States)	
Tsevat J, 1996	79	Human immunodeficiency virus infection				(United States)	

*Nephrology*							
Canadian Group, 1990	80	Chronic renal failure		58	45	(Canada)	
Churchill DN, 1984	81	Chronic renal failure		56	45 (16-77)	(Canada)	
Churchill DN, 1987	82	Chronic renal failure				(Canada)	
Churchill DN, 1991	83	Chronic renal failure		53	60 (20-75)	(Canada)	
Groome PA, 1999	84	Chronic renal failure		68	53 (21-69)	(Canada)	
Laupacis A, 1991	85	Chronic renal failure				(Canada)	
Laupacis A, 1996	86	Chronic renal failure				(United Kingdom)	
Law AV, 1998	72	Chronic renal failure				(United States)	
Moizahn AE, 1996	87	Chronic renal failure			42, 48	(Canada)	
Revicki DA, 1992	88	Chronic renal failure		33	59 (18-75)	(United States)	
Russell JD, 1992	89	Chronic renal failure		74	42	(Canada)	
Sackett DL, 1978	76	Chronic renal failure				(Canada)	
Sesso R, 1996	90	Chronic renal failure		61	43	54% White	
Sesso R, 1997	91	Chronic renal failure		62	49	64% White	
Torrance GW, 1973	77	Chronic renal failure				(Canada)	

*Neurology*							
Ackerman SJ, 1999	92	Amyotrophic lateral sclerosis				(United States)	
Aoki N, 1998	93	Cerebral aneurysm				(Japan)	
Dolan P, 1996	94	Disabled				(United Kingdom)	
Fryback DG, 1993	18	Migrane, Stroke				100% White	
Gage BF, 1996	19	Stroke		86	70	87% White	
Gore JM, 1995	95	Stroke					
Hallan S, 1999	96	Stroke				(Norway)	
Holmes AM, 1997	97	Disabled		41		(United States)	
Kwa VI, 1996	98	Stroke		50	63	(Netherlands)	
Messori A, 1998	99	Epilepsy				(Italy)	
Mushlin AI, 1994	100	Multiple sclerosis			38	(Unites States)	
Patrick DL, 1994	101	Coma, Alzheimer’s disease				(Unites States)	
Richardson J, 1997	102	Disabled				(Australia)	
Samsa GP, 1998	103	Stroke		52	65	(United States)	
Sano M, 1999	104	Alzheimer’s disease		76	46 (22-65)	(United States)	
Shin AY, 1997	105	Arteriovenous malformation		55	37 (18-57)	71% White	
Stavem K, 1998	106	Epilepsy		42	44 (18-67)	(Norway)	
Stavem K, 1999	107	Epilepsy		44	44	(Norway)	
Torrance GW, 1984	108	Disabled				(Canada)	
Ubel PA, 1996	109	Meningioma				(United States)	

*Obstetrics*							
Kuppermann M, 1997	110	Down syndrome, Artificial abortion, Spontaneous abortion		0		86% White	
Kuppermann M, 1999	111	Down syndrome, Artificial abortion			38	76% White	
Saigal S, 1999	112	Low birth weight		23	42	(Canada)	

*Oncology*							
Grann VR, 1998	60	Metastatic disease			38 (22-63)	(United States)	
Grann VR, 1999	61	BRCA1/2 mutations, Metastatic disease		0	33 (20-50)	58% White	
Grunberg SM, 1996	113	Malignant disease		27	56 (23-78)	(United States)	

*Ophthalmology*							
Bass EB, 1997	114	Visual impairment		28	74	(United States)	
Brown MM, 1999	115	Diabetic retinopathy		39	63 (28-87)	95% White	
Fryback DG, 1993	18	Cataract, Glaucoma, Macular degeneration				100% White	
Torrance GW, 1984	77	Visual loss				(Canada)	

*Orthopedics*							
Chang RW, 1996	116	Osteoarthrosis			(22-62)	(United States)	
Chung KC, 1998	117	Carpal tunnel syndrome				(United States)	
Fryback DG, 1993	18	Musculoskeletal pain, Gout				100% White	
Gabriel SE, 1999	118	Osteoporosis, Fracture		0	70	(United States)	
Goossens ME, 1999	119	Musculoskeletal disease			42	(Netherlands)	
Kaplan RM, 1979	42	Loss of extremities				(United States)	
Laupacis A, 1993	120	Osteoarthrosis		52	64 (40-75)	(Canada)	
Patrick DL, 1994	101	Musculoskeletal pain			60	(United States)	
Ubel PA, 1996	109	Musculoskeletal disease				(United States)	

*Psychiatry*							
Chouinard G, 1997	121	Schizophrenia				(United States)	
Dolan P, 1996	94	Depression				(United Kingdom)	
Fryback DG, 1993	18	Depression, Sleep disorder				100% White	
Hatziandreu EJ, 1994	122	Depression				(United Kingdom)	
Revicki DA, 1996	123	Schizophrenia		76	49 (20-69)	(United Kingdom)	
Revicki DA, 1997	124	Depression				(United States)	
Revicki DA, 1998	125	Depression		23	42 (18-65)	(Canada)	
Sackett DL, 1978	76	Depression				(Canada)	
Torrance GW, 1984	108	Depression				(Canada)	

*Pulmonology*							
Blumenschein K, 1998	126	Asthma		25	40	(United States)	
Coley CM, 1996	127	Bacterial pneumonia		36	44	(United States)	
Fryback DG, 1993	18	Asthma, Chronic bronchitis, Chronic obstructive pulmonary disease				100% White	
Goodwin PJ, 1988	128	Lung cancer				(Canada)	
Guyatt GH, 1999	129	Chronic obstructive pulmonary disease				(Canada)	
Jaeschke R, 1994	130	Chronic obstructive pulmonary disease		72	68 (55-77)	(Canada)	
Juniper EF, 1997	131	Asthma		58	12 (7-17)	(Canada)	
Kaplan RM, 1979	42	Respiratory failure				(United States)	
Kennedy W, 1995	132	Lung cancer				(Canada)	
O’Brien B, 1994	133	Chronic obstructive pulmonary disease	54		62		(Canada)
Ramsey SD, 1995	134	Respiratory failure	38		44		(United States)
Ramsey SD, 1995	135	Respiratory failure	38		44		(United States)
Rutten van Molken MP, 1995	136	Asthma	56		53 (18-70)		(Netherlands)
Stavem K, 1999	137	Chronic obstructive pulmonary disease	58		57		(Norway)
Tousignant, 1994	138	Sleep apnea syndrome	74		57 (34-73)		(Canada)

*Rheumatology*							
Bakker C, 1994	139	Ankylosing spondylitis, Fibromyalgia	26		42		(Netherlands)
Bakker C, 1994	140	Ankylosing spondylitis	71		44		(Netherlands)
Bakker C, 1995	141	Fibromyalgia	0		43		(Netherlands)
Blumenschein K, 1998	142	Arthritis					(United States)
Ferraz MB, 1994	143	Rheumatoid arthritis	74		41		(Canada)
Fryback DG, 1993	18	Arthritis					100% White
Hopman-Rock M, 1997	144	Arthritis	34		65 (55-74)		(Netherlands)
Lambert CM, 1998	145	Rheumatoid arthritis	28		57 (28-78)		(United Kingdom)
Moore AD, 1999	146	Systemic lupus erythematosus	4		44		(Canada)
O’Brien BJ, 1990	147	Ankylosing spondylitis, Rheumatoid arthritis	45		48		(United Kingdom)
Peeters HR, 1999	148	Rheumatoid arthritis	18		57		(Netherlands)
Thompson MS, 1986	149	Rheumatoid arthritis					(United States)
Verhoeven AC, 1998	150	Rheumatoid arthritis	42		49		(Netherlands)

*Surgery*							
Ashby J, 1994	151	Breast cancer	25				(United Kingdom)
Dranitsaris G, 1999	152	Breast cancer	0		45 (28-66)		(Canada)
Grann VR, 1998	60	Breast cancer			38 (22-63)		(United States)
Grann VR, 1999	61	Breast cancer	0		33 (20-50)		58% White
Hayman JA, 1997	153	Breast cancer	0		53 (25-84)		(United States)
Hutton J, 1996	154	Breast cancer	0				(United Kingdom)
Jansen SJ, 1998	155	Breast cancer	0		(33-82)		(Netherlands)
McLachlan SA, 1999	156	Breast cancer	0		56 (38-77)		(Canada)
McLeod RS, 1995	157	Operation procedures	52		58		(Canada)
Sackett DL, 1978	76	Breast cancer	0				(Canada)
Unic I, 1998	158	Breast cancer	0		38		(Netherlands)

*Urology*							
Bennett CL, 1997	159	Prostate cancer	100				(United States)
Chapman GB, 1998	160	Prostate cancer	100		71		83% African American
Cowen ME, 1998	161	Prostate cancer	100		66		67% White
Krahn MD, 1994	162	Prostate cancer					(Canada)
Kuo RL, 1999	163	Nephrolithiasis	62		46 (15-82)		(United States)
Matchar DB, 1997	164	Prostate cancer			40		(Canada)
Stiggelbout AM, 1994	165	Testicular cancer	100		31 (20-56)		(Netherlands)
Stiggelbout AM, 1995	57	Testicular cancer	100		30		(Netherlands)
Stiggelbout AM, 1996	166	Testicular cancer	100				(Netherlands)

*Miscellaneous*							
Cairns J, 1996	167	Cystic fibrosis	0				
Clarke AE, 1997	168	Gaucher disease	32		47		(Canada)
Cykert S, 1999	169	debility	41		60		(United States)
Dolan P, 1996	94	Discomfort					(United Kingdom)
Fryback, 1993	18	Allergy					White
Lalonde L, 1999	23	No disease					(Canada)
Lalonde L, 1999	24	No disease					(Canada)
Lenert LA, 1997	170	Drug adverse event			47		(United States)
Lundberg L, 1999	171	No disease			20-80+		(Sweden)
Morss SE, 1993	172	Drug adverse event	94		43 (23-73)		(United States)
Torrance GW, 1984	108	Discomfort					(Canada)
Wells KB, 1999	173	No disease	37				(United States)
Wilkinson TJ, 1997	174	Thiamine deficiency	44		73		(New Zealand)

Mean age was measured as whole group. If race was not reported in the article, the country where the utility values were elicited were shown in the parentheses.
